# Disruption of Circadian Rhythm Genes in Obstructive Sleep Apnea Patients—Possible Mechanisms Involved and Clinical Implication

**DOI:** 10.3390/ijms23020709

**Published:** 2022-01-10

**Authors:** Agata Gabryelska, Szymon Turkiewicz, Filip Franciszek Karuga, Marcin Sochal, Dominik Strzelecki, Piotr Białasiewicz

**Affiliations:** 1 Department of Sleep Medicine and Metabolic Disorders, Medical University of Lodz, 92-215 Lodz, Poland; szymon.turkiewicz@stud.umed.lodz.pl (S.T.); filip.karuga@umed.lodz.pl (F.F.K.); sochalmar@gmail.com (M.S.); piotr.bialasiewicz@umed.lodz.pl (P.B.); 2 Department of Affective and Psychotic Disorders, Medical University of Lodz, 92-215 Lodz, Poland; dominik.strzelecki@umed.lodz.pl

**Keywords:** obstructive sleep apnea (OSA), circadian clock, chronobiology, sleep disruption, hypoxia, polysomnography (PSG)

## Abstract

Obstructive sleep apnea (OSA) is a chronic condition characterized by recurrent pauses in breathing caused by the collapse of the upper airways, which results in intermittent hypoxia and arousals during the night. The disorder is associated with a vast number of comorbidities affecting different systems, including cardiovascular, metabolic, psychiatric, and neurological complications. Due to abnormal sleep architecture, OSA patients are at high risk of circadian clock disruption, as has been reported in several recent studies. The circadian clock affects almost all daily behavioral patterns, as well as a plethora of physiological processes, and might be one of the key factors contributing to OSA complications. An intricate interaction between the circadian clock and hypoxia may further affect these processes, which has a strong foundation on the molecular level. Recent studies revealed an interaction between hypoxia-inducible factor 1 (HIF-1), a key regulator of oxygen metabolism, and elements of circadian clocks. This relationship has a strong base in the structure of involved elements, as HIF-1 as well as PER, CLOCK, and BMAL, belong to the same Per-Arnt-Sim domain family. Therefore, this review summarizes the available knowledge on the molecular mechanism of circadian clock disruption and its influence on the development and progression of OSA comorbidities.

## 1. Master Circadian Clock and Influence of Light–Night Cycle

The circadian clock is a complex, hierarchical timing system whose molecular elements are located in nearly every body cell. They are under the control of the master circadian pacemaker located in the suprachiasmatic nucleus (SCN) of the hypothalamus [1], which features a very similar molecular machinery to the peripheral circadian clock in the body cells. The most important function of SCN is collecting external cues from the retina, which enables the synchronization of the circadian clock with the light/dark cycle, and determines its duration over 24 h rhythm. The master clock generates a pronounced circadian rhythm of neuronal firing frequency, which, through a variety of direct and indirect output pathways, synchronizes other cells throughout the body [2]. Signals from the retina are transmitted by neurons from the retinohypothalamic tract, which axons project to the SCN, where they stimulate neurons by releasing glutamate and pituitary adenylate cyclase-activating polypeptide (PACAP) [3,4], which is a modulating protein [5]. Glutamate acts on N-methyl-D-aspartate receptors (NMDAR), which leads to signal transmission by increasing intracellular calcium and cyclic adenosine monophosphate (cAMP) synthesis in SCN cells [2], which in turn activates kinases, such as calcium/calmodulin-dependent protein kinases (CamK), mitogen-activated protein kinases (MAPK) or protein kinase A (PKA) [6] and phosphorylates cAMP-responsive element-binding protein (CREB) [7]. Phosphorylated CREB is an active transcription factor, which binds to calcium/cAMP regulatory elements (CREs) in promotors of repressors genes, including Per1 and Per2 [8], and stimulates their transcription.

Futhermore, the major circadian clock is autonomous to some extent, generating circadian rhythms by neuronal firing non-dependent from external stimuli [9]. SCN neurons are heterogenic and they differ in their pacemaking ability, neuropeptide expression, and response to environmental timing cues, as well as the rhythms they control [6].

## 2. Molecular Mechanism of the Circadian Clock

**Figure 1:**
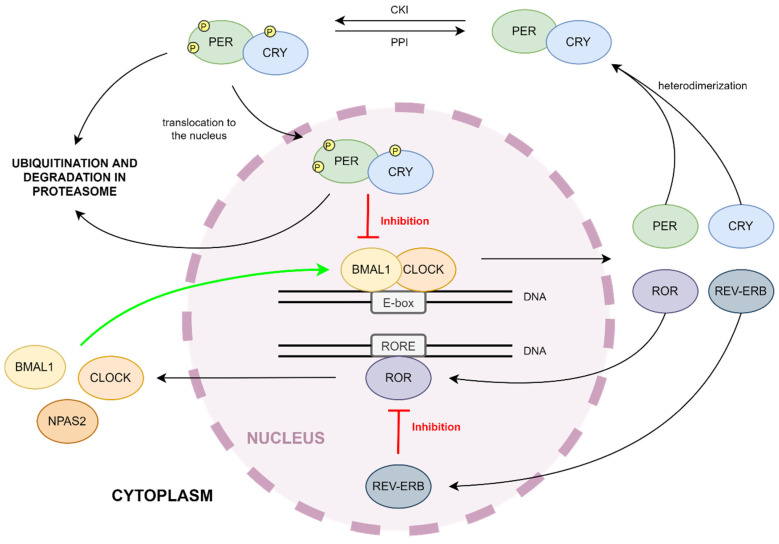
Circadian clock mechanism. BMAL1 and CLOCK are basic helix-loop-helix (bHLH)-PAS transcription factors, whose heterodimer transcripts a large number of genes, such as Per, Cry, Rora, Rev-Erb. PER and CRY are circadian clock repressors. They bind to each other and the PER:CRY complex undergoes phosphorylation, which enables translocation into the nucleus, where it can act as a repressor of BMAL1:CLOCK-dependent transcription. The second feedback loop of the circadian clock consists of two proteins, ROR and REV-ERB. ROR belongs to the same bHLH transcriptor factor family. ROR binds to RORE promotor sequence and transcripts circadian activators. The expression of ROR inhibitor (REV-ERB) occurs at the same time as ROR.

The mammalian circadian clock is based on a transcriptional negative feedback loop between activators and repressors [10], whose function is regulated by kinases and phosphatases [11]. The description of the respective genes is in Table 1. Furthermore, Aryl hydrocarbon receptor nuclear translocator-like (BMAL1) and clock circadian regulator (CLOCK) genes encode subunits of the heterodimeric basic helix-loop-helix PER-ARNT-SIM (bHLH-PAS) transcription factor [12]. BMAL1:CLOCK recognizes E-box motifs (5′CACGTC-3′) in promotors of targeted genes including repressors, and lead to their transcription [13,14]. Among repressors, there are families of Period (Per1, Per2, Per3) and Cryptochrome (Cry1, Cry2) genes [13]. Their protein products heterodimerize in the cytoplasm. PER:CRY undergoes phosphorylation by casein kinases (CKIδ and CKIε) and translocation to the nucleus, where the complex can act as an inhibitor of BMAL1:CLOCK-dependent transcription [1,2] (see Figure 1). The cytoplasm and nucleus level of circadian clock repressors is regulated by the E3 ubiquitin ligase complex (SCF-Fbxl3 complex) and proteasome-dependent pathways of protein degradation [15]. The oscillation of repressors levels conditions the cyclic transcription of circadian clock-controlled output genes and the regulation of behavior [16], lipid, glucose, and redox metabolism [17], sleep [18], body temperature [19], and blood pressure [20], endocrine [16], immune [21] or cardiovascular [22] function.

Nuclear retinoid-related orphan receptors ROR (α, β, γ) are transcription factors from the orphan nuclear receptor family, which bind to ROR response elements (RORE) in the promotors of various genes [23]. The role of RORα in the circadian clock is to regulate the transcription of mainly the BMAL1 gene [24], but also CLOCK, NPAS2 (neuronal PAS domain protein 2, a paralogue of CLOCK; creates a complex with BMAL1; BMAL1:NPAS2 has the same function as BMAL1:CLOCK) [25], and CRY1 (see Figure 1). The BMAL:CLOCK complex, as a transcription factor, increases the expression of RORα, as well as its inhibitors, such as the Orphan nuclear receptor REV-ERBα. REV-ERBs are a group of two DNA binding protein isoforms, α and β. They bind to RORE in a promotor of BMAL1 and prevent RORα activity [24]. Both factors are part of the second feedback loop in the molecular mechanism of the circadian clock (see Figure 1). This feedback loop drives rhythmic changes in BMAL1 transcription and introduces a delay in CRY1 mRNA expression that offsets it from genes regulated strictly by BMAL1:CLOCK. While rhythmic changes in BMAL1 abundance are not required to drive the activators–repressors loop, the ROR/REV loop-induced delay in CRY1 expression is critical for proper circadian timing [1]. The connection of both feedback loops ensures robustness against noise and environmental perturbations and keeps proper circadian timing [26]. Interestingly, PER2 can enhance BMAL1 expression by RORα even though it binds to REV-ERBα [27].

## 3. Impact of Kinases and Phosphatases on the Circadian Clock

The most important kinases regulating the circadian clock are the casein kinases, CK1 and CK2. Seven distinct genes are encoding CK1 isoforms, but only δ and ε influence circadian clock proteins. CK1 acts on PERs, CRYs, and BMAL1. Various isoforms phosphorylate these proteins in different locations, which exerts an impact on the generated effect. The phosphorylation of circadian clock proteins by CK1δ leads to their stabilization. By contrast, CK1ε phosphorylation conduces to proteasome degradation. CK2 acts on PER2, but there is inconsistent information about its effect. The phosphorylation of BMAL1 by CK2 promotes transport to the cell nucleus [10]. Hirota et al. also found that CK1α-dependent phosphorylation can promote the proteasome degradation of PERs [28].

Glycogen synthase kinase 3β (GSK3β) is another kinase, which phosphorylates REV-ERBα in the mammalian circadian clock. It leads to the stabilization and acceleration of the inhibiting function of REV-ERBα on BMAL1 expression. Other targets of GSK3 are BMAL1, CLOCK, and CRY2. Their phosphorylation by GSK3β destabilizes them, promoting proteasome degradation. Adenosine monophosphate-activated protein kinase (AMPK) also acts as a destabilizer of CRY1 [11].

Little is known about the role of phosphatases in the circadian clock. In mammalians, the effects of PP1 and PP5 seem to be significant. PP1 acts on PER2 and stabilizes it, while PP5 activates CKIε by dephosphorylation [11].

## 4. Possible Molecular Mechanisms in OSA

Obstructive sleep apnea (OSA) is a common chronic sleep-related breathing disorder characterized by recurrent pauses in breathing, which are caused by the collapse of the upper respiratory tract [29]. The prevalence of moderate-to-severe OSA in the general population reaches up to 50% in men and 23% in women, and the risk of OSA development increases with advancing age, male sex, and higher body mass index (BMI) [30]. As a consequence, hypopneas and apneas lead to intermittent hypoxia (IH) [31], which is mediated, among others, by key factors in oxygen metabolism, such as hypoxia-inducible factors (HIFs). HIFs are heterodimeric complexes, which consist of two subunits: α (HIF α) and β (HIF β) [32]. Both subunits belong to the basic helix-loop-helix PER-ARNT-SIM (bHLH-PAS) factor family (the same as BMAL1 and CLOCK), which are constitutively produced in cells [32]. Subunit α is oxygen-sensitive [33]. During normoxia, HIF α undergoes hydroxylation and ubiquitin-dependent degradation, but in hypoxic conditions, it is stabilized and heterodimerizes with subunit β and p300 [34]. The formed complex is transported to the nucleus, where it functions as an active transcription factor [35].

Hypoxia is closely related to circadian clock disruption. Addamovich et al. studied daily rhythms in oxygen and carbon dioxide in mice. They found changes in the levels of both gases in Per1^−/−^ mice during the dark phase compared with their wild-type counterparts [36]. Moreover, clock gene expression was altered and the clock was phase-shifted. Another study connected HIF-1α with synchronizing cellular clocks and circadian gene expression. Short time-spans of decreased oxygenation caused the acceleration of the adaption to jet-lag [37]. Manella et al. noted that the response to hypoxia is time-dependent: different mechanisms may play the main role at different times during the day [38]. They also found complete abrogation to hypoxia in Per1,2^−/−^ mice and a lower expression of circadian clock components.

**Figure 2:**
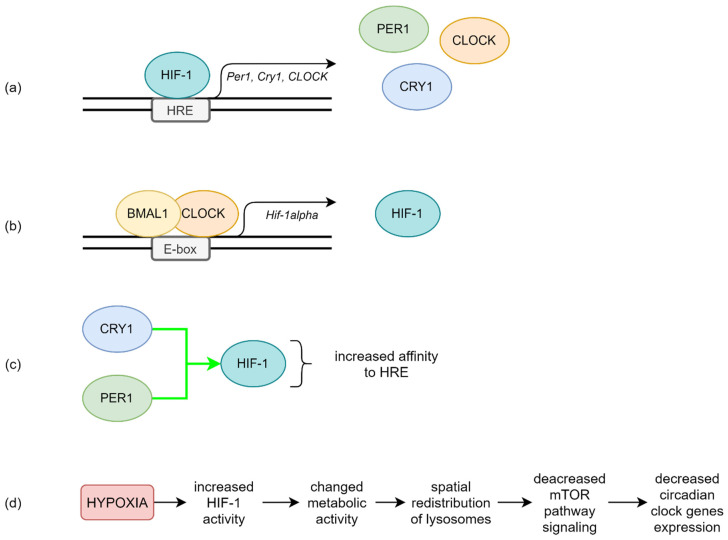
The possible molecular regulation mechanism between HIF-1 and circadian clock proteins. (**a**) HIF-1 can bind to HRE in promotors of circadian clock genes such as Per1, Cry1, and CLOCK. (**b**) Transcriptional activity of BMAL1:CLOCK leads to increased HIF-1 expression. (**c**) CRY1 and PER1 increase HIF-1 affinity to HRE, enhancing HIF-1 activity. (**d**) Influence of hypoxia-dependent acidification on circadian clock genes expression.

The relationship between hypoxia and the circadian clock is not clear, but it is most likely to be bidirectional. As outlined above, hypoxia disrupts the expression of circadian rhythm genes [39]. The main interaction mechanism is probably mutual transcriptional regulation between HIF-1 and BMAL1:CLOCK. The Per1, Cry1, and CLOCK genes feature E-box-like hypoxia response elements (HRE) in their promotors, so they can be targets for HIF-1 [40] (see Figure 2a). Chilov et al. found increased levels of PER1 and CLOCK proteins in mouse brain cells with hypoxia [41]. Additionally, the HIF-1α gene promotor has an E-box sequence, the target of circadian clock activators complex [42] (see Figure 2b). Kobayashi et al. found that PER2 mediates activation of HIF-1α by increasing its affinity to HRE in the study on cell cultures [43] (see Figure 2c). Moreover, HIF-1α accelerates Per2 expression [42].

HIF-1 and circadian clock proteins present a relationship in Obstructive Sleep Apnea patients and their pathways are connected [42,44,45]. In a multivariate general linear model featuring a concentration of all the circadian clock proteins as dependent variables, evening HIF-1α protein level was the only significant covariant (*p* = 0.025). Positive correlations between evening PER1, CRY1, CLOCK, and evening HIF-1α protein levels in patients with OSA have been reported [45]. All the protein levels were measured using ELISA assay. Similar outcomes were obtained among diabetes mellitus type 2 patients [46], patients with hepatocellular carcinoma [34], or varicose lesions [47]. However, early reports suggest that one-night effective continuous positive airway pressure (CPAP) treatment does not affect the level of HIF-1α in OSA patients [48,49].

It emerged that CLOCK and HIF-1α cooperate to induce vasopressin expression in the suprachiasmatic nucleus [50] or reprogram glucose metabolism in hepatocellular carcinoma cells [51]. Both of them bind with MOP3 to enhance transcriptional activity, as does HIF-2α [52]. Peek et al. found that BMAL1 expression disruption leads to an increased level of HIF-1α and the overexpression of its metabolic targets: prolyl hydroxylase 3 (PHD3), vascular endothelial growth factor (VEGF), and lactate dehydrogenase A (LDHA). Moreover, the genetic stabilization of HIF-1α promotes changes in circadian transcription through the heterodimerization of both proteins [53]. The HIF-1α:BMAL1 complex can also increase the expression of PER2 the same way as HIF-1α:ARNT (aryl hydrocarbon receptor nuclear translocator) and CLOCK:BMAL1 complexes [54]. Another study has shown that BMAL1 gene silencing leads to the decreased expression of HIF-1α [55].

The circadian clock features many output genes, including albumin D-element binding protein (DBP) and E4 binding protein 4 (E4BP4). DBP and E4BP4 are, respectively, positive and negative regulators of the HIF-1β promotor. The disruption of this mechanism and ARNT inhibition are conducive to the destruction of pancreatic islet β-cell, decreased insulin output, and diabetes mellitus development in mice [56].

Walton et al. proposed another mechanism of this complicated relationship based on the hypoxic alteration of metabolic activity mediated by HIF-1α, which leads to acidification and the spatial redistribution of lysosomes (see Figure 2d). Acid prevents mechanistic target of rapamycin kinase (mTOR) localization to the lysosomal surface and its activation, which decreases circadian clock expression [57].

**Figure 3:**
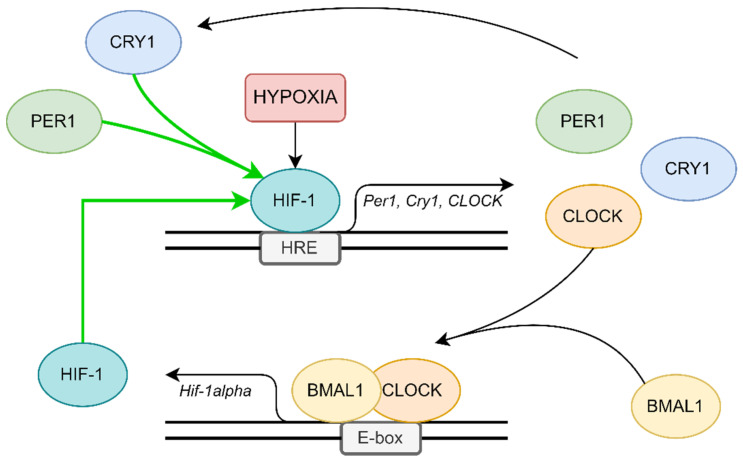
Possible positive feedback loop, which can be responsible for circadian clock disruption in patients.

Based on the results of our study [45] and the above information, it seems that hypoxia mediated by HIF-1 is the most likely mechanism of circadian clock disruption in OSA patients. The first free molecular pathways can interfere with each other. HIF-1 increased the expression of circadian clock genes in the presence of HRE in their promotors and can also intensify the transcription of HIF-1. Moreover, circadian clock repressors aggravate HIF-1 activity. All these create an ordered structure resembling a positive feedback loop (see Figure 3).

## 5. Clinical Implications of Circadian Rhythm Gene Disruption in OSA Patients

**Figure 4:**
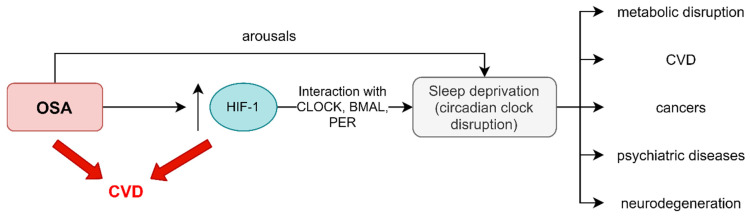
Overlapping of possible complications caused by OSA and circadian disruption. OSA can be both an HIF-1 dependent and an arousal-dependent risk factor for sleep deprivation. HIF-1 overexpression associated with OSA may lead to circadian clock disruption via CLOCK, BMAL, and PER interaction. Disruption of sleep architecture in patients suffering from OSA might be a HIF-1 independent cause of sleep deprivation. This condition can result in an increased risk of a variety of diseases, including metabolic disruption, CVD, cancer, psychiatric diseases, and neurodegeneration. Moreover, both OSA and HIF-1 overexpression may be a risk factor of CVD unrelated to sleep deprivation.

Difficulty in waking up, problems with falling asleep, and daytime sleepiness are the main symptoms of circadian disruption, which is defined as a misalignment between the central circadian clock, located in SCN, and the behavioral cycle. The diagnosis is based on a sleep diary, detailed patient history, and actigraphy [18]. The circadian misalignments are mainly caused by sleep disturbance, jet lag, night shifts, irregular shift work patterns, and dietary alterations [58,59]. Patients with OSA are in a high-risk group of developing disruption of the circadian rhythm. As described earlier, this phenomenon might be explained by the increased level of subunits α of HIF-1 in OSA patients, which is associated with the overexpression of circadian clock proteins, such as PER1 [45,60,61]. This is of great importance in the context of various complications, including the development of metabolic, cardiovascular, psychiatric, and neurodegenerative diseases [49,62,63]. The pathophysiological pathways involved in the development of the aforementioned complications in the group of patients suffering from OSA and/or circadian disruption are not well understood. Moreover, due to the interaction between HIF-1α and the circadian clock, it is difficult to determine whether the complications are caused by the influence of HIF-1 α or independently by the proteins determining circadian rhythms. Another association between OSA and circadian rhythms might be related to arousals interrupting sleep, which can also lead to circadian clock disruption [64] (see Figure 4).

### 5.1. Metabolic Diseases

The circadian clock coordinates and regulates various physiological processes, including metabolism [65], via the central and peripheral clock. The signals between them are transmitted through hormonal, neuronal, and body temperature pathways and their coordination is crucial for circadian alignment [66]. The circadian clock is involved in a temporal separation of opposing processes of catabolism and anabolism. Moreover, it maintains the proper intensity of metabolic cycles during the sleep/wake cycle. In an evolutionary sense, it increases energetic efficiency [59]. The disruption of its function may lead to diabetes mellitus type 2 (T2DM), dyslipidemia, obesity, and metabolic syndrome [46,67,68,69].

Populations with circadian disruption have a twofold increased risk of T2DM development [70]. Clock misalignment is associated with decreased or increased insulin secretion, impaired glucose tolerance, and an alteration of the pancreatic B-cell function [71]. In a study by Scheer et al., 10 healthy adults were exposed to a 10 day protocol. All the subjects were eating and sleeping at all phases of the circadian cycle. This was achieved by scheduling a recurring day that lasted not 24, but 28 h. The test performed during the study revealed decreased leptin levels, increased glucose levels despite increased insulin levels, completely reversed daily cortisol rhythm, and increased mean arterial pressure. Moreover, circadian misalignment caused a postprandial glucose response characteristic of a prediabetic state in 3 out of 8 patients [72]. The risk of metabolic syndrome is also doubled in people suffering from circadian disruption [70]. This phenomenon might be explained by the fac, that between 10 and 30% of genes (depending on the tissue) are characterized by rhythmic expression guided by circadian clock genes. Furthermore, this phenomenon occurs in the case of genes coding various transport systems and metabolic enzymes. Fatty acid transporter sirtuin-1 and albumin D-site binding protein are examples of such transporters. While in the case of enzymes, the enzymes responsible for glucose and lipid metabolism, such as 3-hydroxy-3-methylglutaryl-coenzymeA (HMG-CoA) synthase, acetyl-coenzyme A oxidase, and lipoprotein lipase were affected [73,74].

The risk of developing T2DM and metabolic syndrome in OSA patients is also increased [75]. Mahmood et al. found that the prevalence of T2DM in OSA patients was 30.1%, while in the healthy control group it was only 18.6% [76]. Additionally, not only the prevalence but also the pathophysiology of metabolic complications is similar between OSA patients and patients with disrupted circadian rhythms. In both cases, it might be mediated by HIF-1α and based on impaired glucose tolerance, increased insulin secretion, alteration in B-cell function, and the influence of metabolic enzymes such as acetyl-coenzyme A [49,77]. OSA subjects are also characterized by increased leptin levels [78] and impaired cortisol rhythmicity [79]. Furthermore, the effect of hypoxia may be aggravated by the interaction of circadian components with HIF-1α [43].

**Figure 5:**
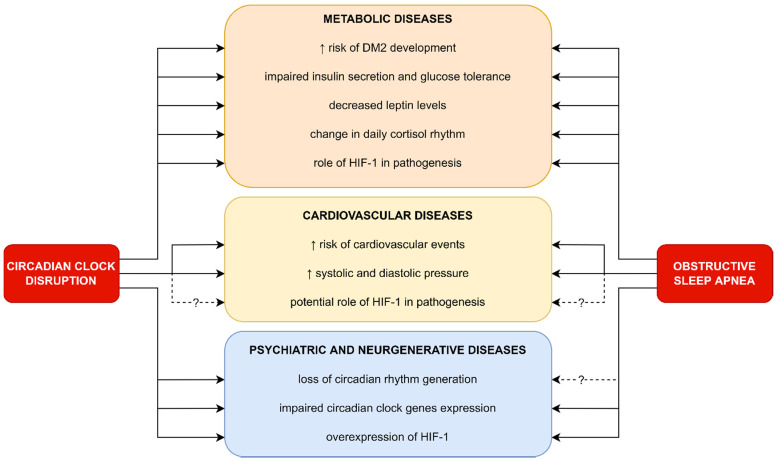
Clinical implications of circadian clock disruption in OSA patients and their similarities in pathogenesis.

Circadian clock disruption is also a potential mechanism of diabetes complications, such as diabetic retinopathy development. The excessive CLOCK-dependent expression of *DEC2* and *VEGF* [80] leads to incorrect neovascularization and in consequence diabetic retinopathy. Moreover, *VEGF* translation is powered by HIF-1α [81] (see Figure 5).

### 5.2. Cardiovascular Diseases

OSA is associated with increased cardiovascular disease (CVD) morbidity and mortality, commonly associated with obesity. The American Academy of Sleep Medicine recommends dietary-induced weight loss and exercise as lifestyle treatment options for OSA. Low-fat diets are recommended for improving OSA severity and weight loss improves OSA severity and the CVD substrate [82]. The best example proving the importance of circadian rhythms in cardiovascular diseases is the fact that myocardial infarction, myocardial ischemia, and sudden cardiac death occur more frequently in the morning than in the evening [22]. This is in contrast to OSA patients, whose peak of cardiovascular risk is in the middle of the night [83,84,85]. Moreover, patients suffering from circadian disruption are at a higher risk of cardiac ischemic events [86]. Other cardiac diseases, such as heart arrhythmias, are also affected by circadian rhythms. The electrical properties of the heart show 24 h variation. Life-threatening arrhythmias, such as ventricular fibrillation, tend to occur in the morning after waking up [87]. The peak of premature ventricular beats detected by continuous Holter monitoring was determined between 6 a.m. and 12 noon [88]. Circadian rhythms also exert an influence on blood pressure. Physiologically, blood pressure dips during the night while resting by 10–20%; in the morning a significant increase in blood pressure occurs, known as the “morning surge”. Blood pressure reaches a peak in the afternoon [20]. Furthermore, there are different circadian patterns among patients with arterial hypertension: dippers, whose blood pressure dips at night; non-dippers, for whom there is no dip of blood pressure at night; and reverse-dippers, who present increased blood pressure during the night [89]. A study by Kitamura et al. revealed that individuals’ blood pressure pattern on the first days of night shift work changed from a dipper to a non-dipper pattern. Furthermore, this phenomenon was reversed after 4 days of night shift work and the dipper pattern was restored [90]. Many mechanisms regulate blood pressure during the 24 h cycle. It is well known that changes in sympathetic nervous system tone are responsible for the “morning surge”. There have been several studies linking the circadian rhythms of blood pressure with kidney and renal sodium homeostasis: higher daytime sodium excretion with urine was associated with the presence of nocturnal dip in the blood pressure [91], aldosteronism was shown to provoke the non-dipping type of hypertension [92], and unilateral nephrectomy was linked with the occurrence of non-dipping blood pressure patterns in patients [93]. In a study by Marques et al., the kidney tissues from hypertensive and normotensive humans were compared. The results showed the upregulation of PER1 in hypertensive humans [94]. Dashti et al. showed the link between single-nucleotide polymorphism in PER1, CRY1, CLOCK, and PER3 genes and systolic blood pressure. A study by Morris et al. revealed that circadian misalignment lasting only 8 days leads to increased systolic and diastolic pressure by 3.0 mmHg and 1.5 mmHg, respectively [95]. Sudden morning increases in blood pressure, heart rate, sympathetic nervous system activity, prothrombic tendency, and vasoconstrictive hormones are thought to be an explanation of myocardial infarction and ischemia peak during the morning [22]. Ischemia leads to hypoxia, which is responsible for HIF-1α protein stabilization in the myocardium and its expression. One of the genes activated by HIF-1α is *VEGF*, which plays an important role in post-myocardial infarction cardiac angiogenesis [96,97]. A study by Koyonagi et al. found that protein Per2 expression reduced the hypoxic induction of HIF-1α-dependent *VEGF* expression [98,99]. Since the expression of Per2 varies during the 24 h cycle, its fluctuation may alternate the cardiac response to ischemia depending on the time of the ischemic episode. However, it is worth mentioning that angiogenesis is a long-term process; therefore, circadian clock disruption must be longer than a day to influence it significantly.

The treatment of cardiovascular diseases in patients with circadian rhythm disruptions is complicated due to the influence of the circadian clock on the pharmacokinetics and pharmacodynamics of drugs [22]. Therefore, chronotherapy based on the understanding of circadian rhythms may help inappropriate drug selection and dosing, and improve the treatment efficiency [100].

In OSA patients, HIF-1 α is also an important regulator of response to hypoxia. There is a vast number of genes regulating the cardiovascular system that are controlled by stabilized HIF1-α, e.g., genes regulating endothelin-1, erythropoietin, and leptin synthesis [101,102]. Therefore, moderate and severe OSA is associated with a significant increase in cardiovascular morbidity [103]. It is also worth mentioning that OSA individuals with comorbid CVD present with higher HIF-1α compared to groups without cardiovascular complications [104]. Due to the cross-talk between clock genes and HIF-1α in OSA patients, the understanding of the pathophysiology of certain cardiovascular complications in OSA and circadian disruption patients is challenging (See Figure 5).

### 5.3. Psychiatric and Neurodegenerative Diseases

Circadian disruption has been found to be related to both psychiatric and neurodegenerative diseases. Some examples include major depressive disorder, bipolar disease, schizophrenia, Alzheimer’s, and Parkinson’s disease [105,106]. Major depressive disorder is characterized by anhedonia, mood alterations, fatigue, changes in appetite and body mass, irritability, and sleep disturbances, including both insomnia and excessive daytime sleepiness [107]. The relationship between major depressive disorder and circadian disruption is bidirectional. On the one hand, depression leads to altered sleep architecture; on the other hand, people suffering from circadian misalignment are more prone to developing a major depressive disorder. In a metanalysis, which included 11 studies, it was determined that night shift workers were at 40% higher risk of developing depression compared with a daytime worker control group [108]. The loss of circadian rhythm generated by the circadian clock is one of the postulated factors leading to depression development. Thus, chronotherapy, which includes sleep deprivation, dark therapy, bright light therapy [109], and others, is a possible treatment option for depression [87]. Interestingly, the expression of HIF-1 α increases three-fold increase in patients suffering from major depressive disorder and 2.5 fold in patients suffering from bipolar disease. Moreover, patients in a remissive state are characterized by significantly lower HIF-1 α compared with patients in a depressive state [86]. The cause of HIF-1 α overexpression in patients suffering from the aforementioned diseases is not clear. One of the postulated factors is oxidative stress caused by an imbalance between the increased production of reactive oxygen species and a relative shortage of antioxidant defense and increased HIF-1 α expression in the protective response to oxidative stress [110,111,112,113], which is similar to the cellular senescence process in OSA patients [114]. Furthermore, increased HIF-1 levels in the brain may improve creatinine metabolism and correlate with a better treatment response to antidepressants [115]. On the other hand, HIF-1 α overexpression may interfere with the genes responsible for circadian clock regulation and modify circadian clockwork. Patients with OSA experience a higher prevalence of depression than healthy controls, even though HIF-1 α offers a protective function against oxidative stress. Therefore, some patients suffering from depression may also improve after CPAP therapy. One of the explanations as to why depression is more frequent in OSA patients might be the circadian disruption caused by arousals and HIF-1 α overexpression [111,116,117].

Bipolar affective disease is a chronic and complex disorder characterized by a combination of different mood episodes including mania, hypomania, and depression. Circadian disruption is a prevalent condition in patients with bipolar disease; however, a recent meta-analysis, which included 42 clinical studies, did not establish an association between circadian disruption and bipolar disease incidence [118]. Despite this, it was reported that bipolar disease can be induced by jet lag in the case of susceptible individuals [105]. Additionally, OSA has been found to be a significant risk factor for bipolar disease. In a study of Kelly et al., 21% of patients with bipolar disease were also suffering from OSA [119]. One of the postulated mechanisms behind this phenomenon was the neurostructural changes seen in decreased gray matter concentration of the amygdala, dorsal lateral prefrontal cortex, hippocampus, cerebellum temporal lobe, caudate lobe, and other areas in the brain of OSA patients [119,120]. However, it is important to remember that many psychiatric disorders, including bipolar disorder, cause an increase in body mass, which in itself may directly contribute to the development of OSA, since an increased body-mass index is a major risk factor for OSA. Moreover, intermittent hypoxemia and circadian cycle disorders with sleep fragmentation in pediatric subjects have shown an association with behavioral and neurocognitive disorders, with reduced school performance. The treatment of OSA problems in children, mainly caused by tonsillar hypertrophy, led to the regression of associated symptoms [121]. This suggests the high probability of a mutual relationship between OSA and psychiatric diseases. The focus in future studies should be on a new generation of drugs, such as aripiprazole, that are not likely to affect the body mass of an individual, increases in which can aggravate OSA problems.

Schizophrenia is another severely disabling mental disorder, characterized by positive and negative symptoms. One of the most prevalent manifestations of this disease is circadian rhythm disruption. It occurs in around 80% of patients [122]. Similarly to the other diseases discussed above, circadian disruption may not only be a sign of disease, but it can also be a cause. Skin fibroblasts, which were isolated from patients suffering from chronic schizophrenia, presented decreased expression of PER2 and CRY1 compared with healthy controls [123]. Moreover, in a study by Sun et al., schizophrenia patients demonstrated altered mRNA levels of PER1/2/3 and NPAS2 in white blood cells compared with a healthy control group [124]. Ying-Ying et al. reported that the prevalence of OSA was increased two-fold in schizophrenia patients compared with a healthy group [125]. Furthermore, in the same study, the hazard ratio adjusted by gender, age, baseline comorbidities, and duration of antipsychotics use was lower for such comorbidities as hypertension, hyperlipidemia, or even diabetes compared with the presence of schizophrenia (HR = 1.61, HR = 1.55, HR = 1.53, and HR = 1.97, respectively) [125]. The interaction between HIF-1 α and CLOCK genes and CLOCK gene alterations in schizophrenia patients seem to be among the possible causes of this prevalence.

Neurodegeneration is any pathological condition in which the nervous system loses its structure or function, or both. Due to increased global life expectancy, the prevalence of neurodegenerative diseases is growing gradually. The disruption of sleep/wake cycles is among the earliest manifestations of these diseases. Moreover, circadian rhythm disruption may be a cause of the neurodegeneration process. For example, the beta-amyloid peptide, which is linked with Alzheimer’s disease, is regulated by the rhythmically expressed presenilin-2 gene in SCN [106]. Additionally, the presenilin-2 gene is regulated in peripheral tissues via CLOCK and BMAL1 [126]. No experimental studies have yet determined that any alteration to clock genes affects presenilin-2 brain expression. Furthermore, a study by Gu et al. found that certain single-nucleotide polymorphisms of PER1 and BMAL1 are associated with an increased risk of Parkinson’s disease. Breen et al., similarly to Cai et al., found that the expression of BMAL1 was decreased in patients suffering from Parkinson’s disease [127,128]. In a study by Ping-Song et al. on 11,664 patients, it was discovered that patients with sleep apnea demonstrated a 1.85-fold higher risk of Parkinson’s disease development compared with the control group [129]. In another study, patients suffering from OSA demonstrated a 2.17-fold higher risk of developing Alzheimer’s disease than no-OSA patients [130]. Such a significant incidence of OSA in patients with neurodegenerative diseases suggests that the association of HIF-1 α and proteins regulating circadian genes may play a substantial role [131]. Surprisingly, HIF-1 α is considered a neuroprotective factor and its activation might play a role in the future treatment of neurodegenerative disorders. In addition, a trial on patients suffering from Alzheimer’s disease with OSA revealed improved cognition in the CPAP-treated group [132,133]. This suggests the possible advantageous effects of the treatment not only on baseline OSA but also on its psychiatric and neurogenerative comorbidities (see Figure 5).

## 6. Conclusions

Due to the pathophysiological association between OSA and circadian rhythm disruption, there seem to be overlapping risk factors for metabolic, cardiovascular, and neurological diseases. Available research proposes a molecular mechanism responsible for these processes. Screening for OSA and, eventually, CPAP therapy might improve the treatment outcome in the selected group of patients with concomitant circadian disruption and certain metabolic, cardiovascular, and neurological diseases. Limited knowledge of the responsible mechanisms limits the possible implementation of more personalized and complex treatment for OSA patients focused on their multiple comorbidities.

## Figures and Tables

**Table 1:** Basic information about main circadian clock proteins [22].

Protein Name	Gene Location on Chromosome	Size (Da)	Size (Amino Acids)	Circadian Clock Function
PER1	17p13.1	136,212	1290	repressor
PER2	2q37.3	136,579	1255	repressor
PER3	1p36.23	131,888	1201	repressor
Cry1	12q23.3	66,395	586	repressor
Cry2	11p11.2	66,947	593	repressor
BMAL1 (ARNTL)	11p15.3	68,762	626	activator
CLOCK	4q12	95,304	846	activator
RORα	15q22.2	58,975	523	regulator
REV-ERBα	17q21.1	66,805	614	regulator

## Abbreviations

AMPK	adenosine monophosphate-activated protein kinase
bHLH-PAS	basic helix-loop-helix PER-ARNT-SIM
BMAL1/ARNTL	brain and muscle ARNT-like 1/aryl hydrocarbon receptor nuclear translocator like
CamK	calcium/calmodulin-dependent protein kinases
cAMP	cyclic adenosine monophosphate;
CKI	casein kinases
CLOCK	clock circadian regulator/circadian locomotor output cycles protein kaput
CPAP	continuous positive airway pressure treatment
CREB	phosphorylate cAMP-responsive element-binding protein
Cry2	cryptochrome 2
CVD	cardio-vascular disease
DBP	albumin d-element binding protein
E4BP4	E4 binding protein 4
E-box	enhancer box; GSK3β—Glycogen synthase kinase 3β
HIF	hypoxia inducible factor
HMG-CoA	3-hydroxy-3-methylglutaryl-coenzyme A synthase
HRE	hypoxia response element
IH	intermittent hypoxia
LDHA	lactate dehydrogenase A
MAPK	mitogen-activated protein kinases
mTOR	mechanistic target of rapamycin kinase
NMDAR	N-methyl-d-aspartate receptors
NPAS2	neuronal PAS domain protein 2
OSA	obstructive sleep apnea
PACAP	pituitary adenylate cyclase—activating polypeptide
PER1	period protein 1
PER2	period protein 2
PER3	period protein 3
PHD3	prolyl hydroxylase 3
PKA	protein kinase A
PP1	protein phosphatase 1
PP5	protein phosphatase 5
REV-ERBα	nuclear receptor subfamily 1 group D member 1
ROR	RAR-related orphan receptor
RORE	ROR response elements
RORα	Nuclear retinoid-related orphan receptors α
SCF-Fbxl3	E3 ubiquitin ligase complex
E3	ubiquitin ligase complex
SCN	suprachiasmatic nucleus
T2DM	diabetes mellitus type 2
VEGF	vascular endothelial growth factor
